# Age Patterns in Dual‐Cycle Identity Processes and Their Associations With Life Satisfaction

**DOI:** 10.1111/jopy.70001

**Published:** 2025-06-26

**Authors:** Joshua A. Weller, Elisabeth L. de Moor, Theo A. Klimstra

**Affiliations:** ^1^ Centre for Decision Research and Department of Analytics, Technology, and Operations Leeds University Business School Leeds UK; ^2^ Department of Developmental Psychology Tilburg University Tilburg the Netherlands; ^3^ Department of Child Study & Human Development Tufts University Medford Massachusetts USA

**Keywords:** age differences, gender differences, identity processes, life satisfaction, lifespan psychology

## Abstract

**Objective:**

Identity development research often applies the identity status approach, which distinguishes different dimensions of identity‐relevant commitment levels and exploration behavior. However, age differences in these dimensions have mostly been examined in adolescence and young adulthood, leaving questions about their variation across the adult lifespan. Additionally, associations between identity and life satisfaction have been equally understudied in adult populations.

**Method:**

We examined these questions in a large, nationally representative U.K. sample (*N* = 3869; age range 18–97). Identity processes were measured using an abbreviated Dimensions of Identity Development Scale. After invariance testing by age groups, we examined age differences across identity dimensions: Commitment and Exploration (depth, breadth, ruminative).

**Results:**

Older individuals reported lower scores on all exploration dimensions until late adulthood. However, though no age differences in commitment were observed between early and middle adulthood, less commitment was reported from middle to late adulthood. Additionally, commitment and exploration in depth were consistently positively associated with life satisfaction, whereas ruminative exploration negatively predicted life satisfaction, with stronger associations appearing in later adulthood.

**Conclusions:**

These findings demonstrate the feasibility of studying identity across adulthood from a measurement perspective and highlight how identity dimensions relate to well‐being at different ages.

Identity development primarily starts in adolescence and continues across the entire lifespan. Yet, most research has focused on adolescents and young adults, and much less attention has been devoted to the years beyond young adulthood (Fadjukoff and Kroger 2016). Even so, the life phases after young adulthood each pose their own unique challenges to identity, such as the transition to parenthood (Höfner et al. 2011; Laney et al. 2015) and to retirement (Osborne 2009). In addition, different life phases may put different demands on individuals' identity, which may cause age‐related variation in the adaptiveness of identity processes. To address these issues, the present study focuses on age patterns in identity process and their links with satisfaction across the adult lifespan (i.e., age 18–97) in the United Kingdom (U.K.).

## 1 Identity Across the Lifespan

Following Erikson's (1963) Lifespan model of development, gaining a sense of identity (versus role confusion) is the central developmental task during adolescence. How individuals manage this task is thought to be associated with how they navigate developmental tasks that are more central to adulthood, such as gaining intimacy versus isolation, developing generativity versus stagnation, and attaining ego‐integrity versus despair. Erikson (1950/1993, 261–262) defined identity as “…the accrued confidence that the inner sameness and continuity prepared in the past are matched by the sameness and continuity of one's meaning for others…”. Later work (Marcia 1966) boiled this rather broad definition down to the more specific concept of commitments to important choices in life, such as the choice of a particular romantic partner or career.

However, identity development does not end after having made commitments. Also, the commonly held belief that identity formation stops by the end of adolescence, or “emerging adulthood”, misrepresents Erikson's (1963) work and subsequent research. Instead, identity formation consists of a dynamic process that allows individuals to continuously evaluate and update their commitments across the adult lifespan. Although already emphasized by Erikson (1963), this dynamic nature was further articulated in later works on models of identity.

Besides emphasizing the importance of commitment, Marcia (1966) attempted to capture the dynamic process of identity development by also focusing on exploration processes. Exploration captures the process of taking stock of and learning more about what identity options are available, for instance, in choosing one's romantic partner or career. Depending on their combination of level as well as the timing of commitment formation and exploration, four identity statuses emerge. Despite Marcia's (1966) emphasis on exploration alongside commitment, this model has been critiqued for inadequately explaining identity development. Specifically, the model was thought to be too “static” because even when it would be applied longitudinally, it only provided snapshots of one's current state of identity formation, with there being little information on the developmental process (e.g., Bosma 1985; Grotevant 1987).

Stephen et al. (1992) attempted to address these critiques by emphasizing that there is no “end point” in identity development, but did not change Marcia's (1966) conceptualization otherwise. More recent “dual‐cycle” models of identity (Crocetti et al. 2008; Luyckx et al. 2008) took a different approach by capturing how individuals may go through more formative and more evaluative phases in their identity development. The periods that are described as “formation” are characterized by broadly exploring one's options and eventually making a commitment to an option. More evaluative periods are characterized by exploring the fit of commitments to one's sense of self more deeply and increasing (or decreasing) one's identification with the commitments accordingly (Luyckx et al. 2008). Furthermore, such dynamic models also explain that individuals may move between such cycles at any point during the lifespan. For example, they may negatively evaluate a previously made commitment, consider discarding it, and therefore return to the formation cycle.

Despite the fact that dual‐cycle models are well‐equipped to assess identity formation across the lifespan, research using these models has been mostly conducted in the second and third decades of life. In adolescence and young adulthood, there is some evidence that, on average, youth tend to follow the theoretical predictions by gaining stronger commitments over time (Branje et al. 2021; Meeus 2011). In addition to these trends being observed in longitudinal work, they are also reflected in cross‐sectional age‐group comparisons, with younger adolescents being more likely to have weak commitments and older adolescents and young adults being more likely to have strong commitments (e.g., Kroger et al. 2010; Meeus 1996; Verschueren et al. 2017). It is important to note that, despite these general trends, there is considerable inter‐individual variation, as many youths do not change at all over periods of several years. Also, there is still a considerable number of identity‐diffused (weakly committed, weakly exploring) individuals among college students (Verschueren et al. 2017) and among individuals in their late twenties (Carlsson et al. 2016).

This past work has also emphasized the existence of potential gender differences in the extent to which individuals engage in identity processes. Particularly, although evidence regarding gender differences in identity is inconclusive, there is some work that suggests girls and young women may be more likely to engage in exploration in breadth and depth (Meeus et al. 2010; Verschueren et al. 2017) as well as ruminative exploration (e.g., Crocetti et al. 2011; Luyckx et al. 2008, 2015) than boys and young men. Regarding commitment, there is no consistent evidence on gender differences in either direction.

Although the bulk of research on identity processes related to exploration and commitment focuses on these early life phases, there is a growing literature base examining identity beyond adolescence and young adulthood. This work suggests that considerable identity development occurs after the early formation periods, triggered by a combination of changing external circumstances and internal needs (Kroger 2015). This work has shown that adults in mid‐adulthood were more often in identity statuses characterized by stronger commitments compared to individuals in young adulthood (Whitbourne and VanManen 1996), and that these differences could in part be explained by situational changes during these life phases (e.g., to marriage and parenthood in midlife; Arneaud et al. 2016). Following the importance of these situational changes, Mehta et al. (2020) suggest that the period of 30–45 years (which they refer to as “established adulthood”) may present unique challenges to women, as they potentially navigate conflict between decisions related to work and childbearing during a period when commitments to careers may be strongest and exploration may be lower. Moreover, across time, adult men and women both have been found to predominantly change toward identity statuses characterized by stronger commitments, and this was true regardless of whether the upper age limit was at 30 (Kroger et al. 2010), 50 (Fadjukoff et al. 2016), or 60 years old (Cramer 2004). However, like in earlier life periods, there is also much evidence for stability later in adulthood. Furthermore, these transitions in identity status have been suggested to mostly be driven by an increase in the strength of identity commitments (Pulkkinen and Kokko 2000).

## 2 Measurement Invariance of Identity Dimensions Across the Adult Lifespan

Importantly, some of this literature has made use of identity measures that were originally created with an adolescent and young adult population in mind (e.g., Utrecht‐Management of Commitments Scale; Crocetti et al. 2008). However, psychometric evaluations in older populations are lacking, and it is possible that these instruments do not always capture identity processes in older populations in the same way as in younger populations (c.f., Fadjukoff and Kroger 2016). In fact, it has even been suggested that different identity processes may be at work during the very last stages of life (e.g., readjustment and maintenance of commitments; Kroger 2002). Similarly, psychometric evaluations across the adult lifespan are also lacking for the Dimensions of Identity Development Scale (DIDS; Luyckx et al. 2008), which was used in the present study. The DIDS captures identity commitment and exploration processes in the formation cycle, in which commitment making captures the degree to which commitments to possible life paths are made and exploration in breadth, the extent to which individuals compare alternative commitment options before selecting one. In the evaluation cycle, it captures identification with commitment, which represents the degree to which commitments are perceived as fitting with one's values and as providing certainty and exploration in depth, or the degree to which existing commitments are evaluated on their fit with oneself. In addition, a fifth process captures the ruminative side of exploration, defined as continuous worry and the inability to settle on satisfying identity choices. The DIDS has been mostly used in adolescent and young adult populations, and in line with this, work on its psychometric properties has also been centered in these age groups (e.g., Johnson et al. 2022). This measure has been applied to a variety of age groups, including individuals well beyond the age of 30. However, such studies (e.g., Claes et al. 2018, 2019) tend not to focus on age comparisons and hence did not compare the psychometric properties of the instrument across age groups.

When examining age trends across the lifespan, it is essential to assess measurement invariance across age groups (e.g., Van de Schoot et al. 2012). Currently, there is a lack of literature describing such tests for identity measures beyond adolescence and young adulthood, which leaves it unclear how appropriate it is to compare means on identity dimensions across different age groups. Briefly, establishing measurement invariance of a construct across some variable (e.g., age, gender, etc.) is a vital step to ensure that any comparisons made are not biased by differences in how the groups, or different individuals, understand or respond to the items (see Vandenberg and Lance 2000 for a more detailed review). Invariance testing typically involves multiple steps. How many steps are necessary depends on the goal of the study (Steenkamp and Baumgartner 1998). First, *configural invariance* (i.e., *“*Is the basic factor structure similar across groups?*”*) needs to be assessed to examine whether the same broader construct (i.e., identity formation) is described by the same number of dimensions in different groups. For example, hypothetically, identity formation could be best described by two broad dimensions (e.g., commitment and exploration) in young adolescents, but with more dimensions (e.g., subtypes of exploration) in older age groups. Second, *metric invariance* (i.e., “Are the factor loadings similar across groups?”) is necessary for making sure that correlations of variables of interest (i.e., identity dimensions) with external variables (e.g., psychological well‐being) can be compared across groups. Third, *scalar invariance* (i.e., “Are the item intercepts equivalent?”) is necessary to ensure that mean scores on a variable of interest (e.g., exploration in breadth) can be compared across different groups. Best practices suggest that a latent construct should achieve at least partial scalar invariance (i.e., one intercept of an indicator may be allowed to be freely estimated for each group, provided that a substantial drop in model fit is not observed; Byrne et al. 1989).

Previous research using a rather different, but still somewhat related instrument for the assessment of identity formation (Topolewska‐Siedzik and Cieciuch 2019) did find age‐related measurement invariance^1^. That suggests some evidence for age invariance in the assessment of identity formation, and our (weak) hypothesis is that this would generalize to the instrument we use: the DIDS.

## 3 Identity and Satisfaction With Life

In line with its central developmental role, empirical evidence supports associations between identity processes and well‐being during adolescence. In general, stronger commitments marked by higher scores on dimensional identity measures or identity statuses characterized by high commitment tend to be associated with higher satisfaction with life (e.g., Branje et al. 2021). Associations between exploration and well‐being have been more mixed, reflecting its dual nature. That is, exploration can be stressful in the moment but potentially leads to more satisfying identity choices over time. For example, adolescents who engaged in more exploration before the transition to secondary school reported higher school adjustment in their new school than those who explored less (de Moor and Branje 2023).

Despite their associations with satisfaction with life, commitment, and exploration processes are not inherently positive or negative, but their functionality may rather depend on the context in which they are expressed. For instance, one study suggested that a cognitive style of approaching identity issues that tends to be associated with low engagement in commitment and exploration may be adaptive for adolescents facing economic pressures and poor material conditions (Vosylis et al. 2021). Similarly, it is possible that commitment and exploration may have differential associations with satisfaction with life depending on the life phase that individuals are in. In fact, one study suggested that exploration in breadth (the consideration of different identity options) is positively associated with depressive symptoms among youth in their late twenties, but not in younger age groups (Luyckx et al. 2013). In addition, the negative association of depressive symptoms with commitment making and its positive association with ruminative exploration was somewhat stronger among individuals in their late 20s. The differences reported by Luyckx et al. (2013) were rather small, and their study focused on a set of merged samples from Belgium. Moreover, they only focused on the period from adolescence up to late twenties, leaving it unclear what these associations would look like later in life when social norms around specific identity profiles (e.g., stronger expectations of being committed and not exploring too much) may be stronger. That is, age‐related differences may partly stem from age‐related norms (i.e., how “normal” it would feel to explore). For example, Carstensen (1993, 2021) suggested that older adults experience more positive well‐being relative to middle‐aged adults (e.g., Blanchflower and Oswald 2008; Brim et al. 2019), in part related to de‐prioritizing exploration goals in older age and focusing on more emotional goals. This insight leads to the possibility that lower exploration would be associated with greater well‐being in older age, whereas greater exploration, especially ruminative, would be more strongly negatively associated with life satisfaction.

## 4 Current Study

Because of the historic focus on adolescence and young adulthood in research on identity, it is unclear if and how mean scores of commitment and exploration processes vary across the adult lifespan, and how they are related to satisfaction with life beyond young adulthood. In the current study, we will examine four main questions regarding age patterns using cross‐sectional data covering the adult lifespan (i.e., 18–97) among individuals residing in the United Kingdom.

First, we will examine whether the measure we use (the DIDS; Luyckx et al. 2008) works similarly across the adult lifespan. We test measurement invariance using moderated nonlinear factor analysis (MNLFA; Bauer 2017a, 2017b). Unlike multiple group designs, MNLFA can test measurement invariance using multiple variables simultaneously, and allows for invariance testing across a continuous variable. This is especially important for a variable like age because the traditional multiple group design approach for invariance testing, which employs a clustering of age groups, could obscure potential nuanced differences. Thus, it provides a powerful, flexible, and efficient means to test the invariance of a construct.

Second, we will examine age differences in identity commitment and exploration processes across the adult lifespan. Based on previous research, we hypothesize greater mean scores in commitment across age cohorts (e.g., Pulkkinen and Kokko 2000). Previous research is less conclusive on age‐related changes in exploration, but some research (Topolewska‐Siedzik and Cieciuch 2019) suggests that exploration processes may peak in the twenties and then decrease through the rest of adulthood. Hence, we would hypothesize similar patterns, but with one exception. Topolewska‐Siedzik and Cieciuch's (2019) oldest age group covered ages 49–65, and therefore, they were unable to examine age differences that would potentially be related to retirement. Given that retirement marks a major life transition, and there is evidence for the adaptive role of exploration around earlier transitions (de Moor and Branje 2023), we might anticipate elevated mean scores in exploration dimensions around retirement age.

Third, we will examine whether age differences in identity commitment and exploration are moderated by gender. Although there is some work suggesting there may be gender differences in adolescence and young adulthood (e.g., Crocetti et al. 2011; Luyckx et al. 2008, 2015; Meeus et al. 2010; Verschueren et al. 2017) the findings are inconclusive, and it is not clear to what extent they transfer to later adulthood.

Fourth, we will test the degree to which identity processes are associated with life satisfaction across the lifespan. We hypothesize that the positive association of commitment with life satisfaction that is typically found may be stronger when such strong commitments become more of the norm in a particular age group. Similarly, exploration may be more positively (or, put differently, less negatively) associated with satisfaction with life in life phases in which individuals are exploring more heavily (e.g., 20s and 30s, and again near retirement).

## 5 Method

### 5.1 Participants and Procedure

We recruited participants through a third‐party crowdsourced research firm (Cint) as part of a larger project. To be eligible for this study, a participant had to be 18 years of age or older and a resident of England. Quotas based on U.K. Census estimates for region, age, and gender were established. Contextualizing our study, the U.K. is a highly individualistic culture, is relatively focused on long‐term goals and virtues (e.g., placing value on long‐term planning, versus more present‐based orientations), and is low on uncertainty avoidance (The Culture Factor, n.d.). Half of young adult men start work at age 23, and half of young adult women at age 24 (Office for National Statistics April 8 2024). The average age of mothers at the birth of a child is 31, and 34 for fathers; the average age of mothers at the birth of their first child is 29. Retirement, defined as the date that one can begin to receive a state pension, is set at age 66.

We followed data cleaning procedures specified in the preregistration^2^. We first screened for evidence of careless responding (e.g., taking less than 5 min to complete the survey, evidence of response sets, self‐reporting that they did not carefully or honestly answer the questions). We used the r package “careless” to identify potential outliers, which calculates indices of careless responding (Yentes and Wilhelm 2021), such as maximum longstring values, intra‐individual response variability indices, and Mahalonobis distances that span beyond a gap in the distribution.

There were 7239 survey clicks on the survey invitation, with 4612 of these clicks agreeing to participate after reading the participant information sheet. We removed participants' data who abandoned the survey (*n* = 88), those who showed clear evidence of straight‐lined responding throughout the survey (*n* = 106), and those who self‐stated that they did not respond honestly or carefully (*n* = 54). Continuing to follow our data retention criteria (e.g., completion duration > 5 min, using the r careless package to further identify problematic cases such as low variability/straight‐lining), we removed 495 additional participants. We retained a final sample size of *N* = 3869. Median age was 48 years (range = 18–97), 49.5% men, 49.9% women, 0.5% transgender/non‐binary/preferred to self‐describe, 0.1% did not report^3^. Participants were primarily of white‐U.K. origin (88.4%); 3.0% reported Black/African/Caribbean ethnicity, 0.3% Asian, 2.5% mixed ethnicity, 0.5% reported another ethnicity, and 0.8% did not respond. With respect to highest attained education level, 3.0% did not complete a secondary school education, 33.0% completed secondary school, 19.9% completed vocational, or similar, training, 33.0% report either some university education, or have completed a bachelor's degree, and 11.2% reported some graduate/professional degree.

### 5.2 Measures

#### 5.2.1 Identity Dimensions

Participants completed the Dimensions of Identity Development Scale‐short form, based on an Item‐Response Theory testing (Johnson et al. 2022) of the original DIDS (Luyckx et al. 2008). This measure includes five dimensions: Commitment‐making, identification with commitment, ruminative exploration, exploration in breadth, and exploration in depth. Each dimension was assessed with three items, and participants responded on a 1 (*strongly disagree*) to 5 (*strongly agree*) Likert scale (see Table 1 for summary statistics and reliability estimates)^4^.

**TABLE 1:** Descriptive statistics for variables of interest.

	*α* (*M* interitem *r*)	Mean	SD
Commitment making	0.82 (0.61)	3.39	0.89
Identification with commitment	0.71 (0.55)	3.39	0.84
Exploration in breadth	0.71 (0.45)	3.50	0.77
Exploration in depth	0.59 (0.42)	3.27	0.91
Ruminative exploration	0.77 (0.52)	3.16	0.93
Satisfaction with life	0.83 (0.57)	3.26	0.92

*N* = 3869.

#### 5.2.2 Satisfaction With Life

Participants also completed 4 items^5^ from Diener et al. (1985) Satisfaction with Life scale, also on a 1 (*strongly disagree*) to 5 (*strongly agree*) Likert scale, “I am satisfied with my life”, “the conditions of my life are excellent”, “So far I have gotten the important things I want in life”, and “If I could live my life over, I would change almost nothing”.

### 5.3 Data Analytics Plan

We first conducted correlational analyses in the full sample to test the overall relationships between the identity dimensions, followed by their associations with age and gender. Before testing for age differences, we conducted invariance testing for the identity dimensions across age, which was treated as a continuous variable.

We assessed measurement invariance for age and gender using moderated non‐linear factor analysis (MNLFA; Bauer 2017a, 2017b). MNLFA can simultaneously test a covariate's moderation with an item's factor loading (satisfying metric invariance) and intercept (satisfying scalar invariance), assessing differential item functioning (DIF), which refers to when an item functions differently for different groups, potentially indicating bias (e.g., Bauer 2017a, 2017b; Kolbe et al. 2024). Perhaps even more importantly for the goal of the study, it also allows for treating age as a continuous variable, which allows us to circumvent issues inherent to categorical approaches to age differences. We followed the procedure specified by Bauer (2017a, 2017b), following which we first specified the model for each identity dimension. Then we estimated the model parameters for means and variance, and the impact of the covariates on factor means and variances. In the third step, we tested for DIF for each scale item independently. This involved testing the moderated effect between a covariate (i.e., age) and both the (a) factor loading and (b) intercepts. For partial metric and scalar invariance to be met, at least two invariant parameters of a kind were needed to establish partial invariance (e.g., for partial scalar invariance, we would need at least two of the intercepts to be set equal). Due to the multiple tests being conducted, we applied the Benjamini–Hochberg correction for false discovery rates for each factor, setting the false discovery rate at 0.05 (Benjamini and Hochberg 1995). Finally, we compared Bayesian Information Criterion (BIC) values from the item‐specific DIF models to a model without any specified DIF to determine if the inclusion of the DIF path significantly improved model fit. Item DIF models that showed improvement of ≥ 10 were retained. If a scale had multiple DIFs that improved fit, we respecified the model with both DIF paths. If the identity dimension yielded non‐invariance at either the factor loading or intercept level, follow‐up testing was conducted to test boundaries of measurement invariance by progressively decreasing the age range of the measurement test by 5 years until significant non‐invariance was met (i.e., analyses from age 18–77, then 18–72, and so on). When non‐invariance was discovered, the analyses continued using the upper age of the last invariant model as the baseline (for a similar approach, see Klimstra et al. 2020).

Our MNLFA tests also include age, gender, and age × gender as predictors of factor scores, which allow for the simultaneous estimation of these effects for each identity dimension, minimizing the number of inferential tests, and thus reducing the likelihood of Type 1 errors when examining age differences. Finally, we examined the degree to which identity dimensions were associated with life satisfaction. Like the age differences analysis, we tested the degree to which the age groups differed in life satisfaction, and whether gender moderated this effect with a linear regression analysis.

## 6 Results

### 6.1 Correlations Between Identity Processes, Age, and Gender

We first examined the degree to which the DIDS scales were associated with each other. Consistent with past research (Luyckx et al. 2008), we observed strong correlations between commitment‐making and identification with commitment scales (Table 2). Also, we found that the exploration scales (breadth and depth) were moderately correlated with each other and the commitment scales. Ruminative exploration was positively associated with the two other exploration dimensions and weakly inversely related to the commitment variables.

**TABLE 2:** Correlations between demographics and DID‐SF scales.

	1	2	3	4	5	6	7
1. Age	—						
2. Gender (men = 0)	−0.28**	—					
3. Commitment making	−0.06**	−0.01	—				
4. Identification with/commitment	−0.08**	0.00	0.69**	—			
5. Exploration in breadth	−0.31**	0.10**	0.44**	0.37**	—		
6. Exploration in depth	−0.33**	0.13**	0.41**	0.41**	0.57**	—	
7. Ruminative exploration	−0.42**	0.16**	−0.08**	−0.07**	0.48**	0.39**	—

** *p <* 0.01.

Age was inversely associated with the exploration‐oriented variables, ranging from −0.31 to −0.42. In contrast, age only showed very modest associations with the two commitment variables. In a similar vein, gender was not correlated with commitment variables, but women, on average, reported higher on exploration‐oriented identity dimensions.

#### 6.1.1 Measurement Invariance of the DIDS‐SF Scales

Because invariance testing can only be conducted on scales with more than two indicators, we focused our analyses on exploration in breadth, ruminative exploration, and a composite commitment variable because of the high correlation between the two commitment scales in this study (*r* = 0.69; also observed in prior research, Klimstra et al. 2013; Luyckx et al. 2008)^6^. Table 3 shows the results of the invariance testing for these DIDS‐SF scales.

**TABLE 3:** Measurement non‐invariance identified by MNLFA for Identity Dimensions.

Scale	DID‐SF Item				
*Exploration in breadth*	*Item 1*	*Item 4*	*Item 10*		
Factor loading (SE)	0.80** (0.05)	**0.62** ** (**0.04**)	**0.69** ** (0.04)		
Loading DIF (SE)					
Age	0.05** (0.02)	−0.06** (0.02)	0.04 (0.02)		
Gender	0.01 (0.04)	0.07 (0.03)	−0.03 (0.04)		
Intercept DIF (SE)					
Age	0.02 (0.02)	0.**18** ^**^ **(0.02)**	**−0.17** ^**^ **(0.02)**		
Gender	−0.03 (0.03)	−0.03 (0.03)	0.07 (0.03)		
Model BIC (baseline: 29387.11)	29,407.69	**29,282.27**	**29,286.95**		
*Ruminative exploration*	*Item 6*	*Item 7*	*Item 9*		
Factor loading (SE)	0.81** (0.04)	0.87** (0.04)	0.65** (0.03)		
Loading DIF (SE)					
Age	0.01 (0.02)	−0.02 (0.02)	0.**07** ^**^ **(0.02)**		
Gender	−0.03 (0.03)	0.01 (0.03)	0.04 (0.03)		
Intercept DIF (SE)					
Age	**−0.11 (0.06)**	0.**20 (0.03)**	**−0.08 (0.02)**		
Gender	−0.03 (0.03)	−0.01 (0.04)	0.02 (0.04)		
Model BIC (baseline: 31199.27)	31,196.69	**31,153.35**	31,197.10		
*Commitment composite*	*Item 2*	*Item3*	*Item 5*	*Item 8*	*Item 11*
Factor loading(SE)	0.87** (0.04)	0.94** (0.04)	0.86** (0.04)	0.76** (0.04)	0.69** (0.04)
Loading DIF (SE)					
Age	0.01 (0.01)	−0.03 (0.02)	−0.04 (0.02)	0.04 (0.02)	0.03 (0.02)
Gender	−0.01 (0.03)	0.02 (0.03)	−0.01 (0.03)	0.02 (0.03)	−0.03 (0.03)
Intercept DIF (SE)					
Age	**−0.12** ** **(0.01)**	0.**07** ** **(0.01)**	0.**07** ** **(0.01)**	**−0.06** ** **(0.01)**	0.03 (0.04)
Gender	0.02 (0.03)	0.00 (0.02)	−0.03 (0.07)	0.00 (0.03)	0.02 (0.03)
Model BIC (baseline: 46,633.09)	**46,558.416**	**46,623.971**	**46,619.485**	46,634.5	46,656.61

*Note:* Parameter values in bold survived the sensitivity analysis for false discovery. BIC values < 10 compared to the baseline (no DIF model) in bold.

** *p* ≤ 0.01.

##### 6.1.1.1 Exploration in Breadth

We found some evidence for age‐related moderation of factor loadings on two items, suggesting metric non‐invariance (i.e., unequal factor loadings across ages), as well as significant DIF effects for two items, suggesting scalar non‐invariance: Older participants were more likely to endorse “I think about different things I might do in the future”, and less likely to endorse “I am thinking about different lifestyles that might be good for me.” Given these issues, we adopted the alternative strategy of examining the age ranges in which invariance could be obtained. Our analyses suggested partial scalar invariance from ages 18 until 47 years (controlling for item 4 DIF). To maintain continuity, we began the next set of MNLFAs with individuals 43 years and older (i.e., the starting age of the last 4‐year interval in the prior analysis). We found full metric and partial scalar invariance (also accounting for Item 4 DIF) from 43 to 72 years, and then full metric and scalar invariance from age 68 until the maximum age in the sample (see Table S1 for these models).

##### 6.1.1.2 Ruminative Exploration

The MNLFA yielded no significant gender interactions and only one age interaction, for the factor loadings of each item. We did find some significant age‐related linear moderation of the intercepts. Age was positively associated with endorsement of the item “I keep looking for the direction I want to take in my life” (item 7), but negatively associated with “I worry about what I want to do with my life”(item 6). However, BIC comparisons of DIF models to baseline suggested that only the inclusion of item 7 DIF would improve fit, and thus, only retained the DIF of this item for our analyses in the full sample.

##### 6.1.1.3 Commitment Composite

Because the commitment composite scale had five indicators, we first fit a one‐factor CFA model, which achieved acceptable model fit (Table S2) The configural model, which was tested across age groups, clustered in 4‐year spans^7^, showed acceptable goodness of fit across the entire sample, with CFI, TLI, and SRMR values being in the acceptable range, though RMSEA values indicated poorer model fit. We then used MNLFA in the overall sample to test metric and scalar invariance for age and gender simultaneously. Our results revealed no metric non‐invariance for either age or gender. Gender was not associated with DIF on any items. However, we did find age‐related DIF for four of the items: Two items were endorsed more in younger participants (Item 2, “I have plans for what I am going to do in the future” and Item 8 “My future plans give me self‐confidence”), whilst two were endorsed more in older participants (Item 3 “I know which direction I am going to follow in my life” and Item 5 “I have chosen what I am going to do with my life”). Examination of BIC values suggested that accounting for DIF for only 3 items improved model fit compared to the overall model (items 2, 3, and 5), and thus, we consider this the final model.

In sum, we found at least partial scalar invariance across wide age ranges for each of the three dimensions. Although there were some differences in the range across dimensions, each dimension was invariant from age 18 until 42, through adulthood until at least 72 years of age, and then in later life.

### 6.2 Age and Gender Differences in Identity Processes

**FIGURE 1:**
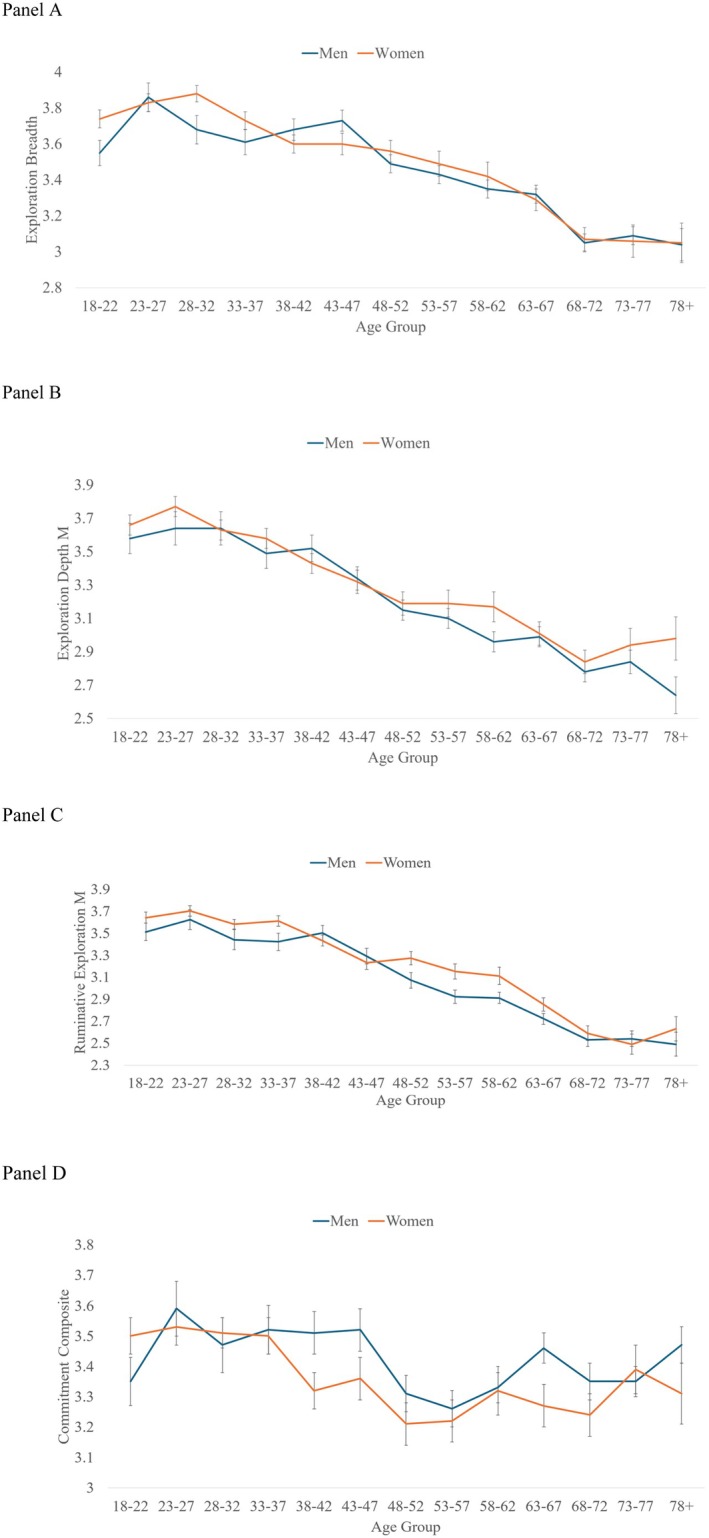
(A–D) Age differences in identity dimensions, by gender. Panel A= Exploration in Breadth, Panel B= Exploration in Depth, Panel C= Ruminative Exploration, Panel D= Commitment Composite. For exploration in breadth, age‐related invariance was found from ages 18–47, 43–72, and age 68–97. Please see Supporting Information for details.

The MNLFAs allowed us to simultaneously examine age and gender differences in each DIDS‐SF dimension, as well as potential non‐linear age effects (i.e., quadratic and cubic effects) and the degree to which gender moderates these effects. Mean plots based on four‐year intervals are presented in Figure 1 for illustrative purposes.

#### 6.2.1 Exploration in Breadth

Because the exploration in breadth scale showed non‐invariance across the adult lifespan, we present the age differences across three different periods (see Table 4). From 18 to 47 years, we observed a significant Age × Gender effect, in which women reported greater exploration in breadth earlier in adulthood, but less than men at the end of this period. In the second analysis (ages 43–72), we found a significant effect for age, irrespective of gender, in which older participants in this age group reported lower exploration. In the analysis with individuals aged 68 and above, we observed no significant age, gender, or interaction effects.

**TABLE 4:** MNLFA estimates of age and gender effects predicting identity process factor scores.

Predictor	*B*	SE
**Exploration in breadth**		
*18–47 years*		
Age	0.16	0.10
Gender	0.10	0.06
Age × Gender	−0.16**	0.06
*43–72 years*		
Age	−0.17**	0.09
Gender	0.01	0.06
Age × Gender	−0.08	0.06
*68 years and older*		
Age	−0.02	0.18
Gender	−0.06	0.15
Age × Gender	−0.06	0.11
**Ruminative exploration**		
Age	−0.62**	0.07
Gender	0.12**	0.04
Age × Gender	0.04	0.04
**Commitment composite**		
Age	0.02	0.06
Gender	−0.07	0.04
Age × Gender	−0.08*	0.04

* *p* < .05.

** *p* < .01.

#### 6.2.2 Exploration in Depth

Because this scale only included two items, we could not test for measurement invariance. Taking a conservative approach given the non‐invariance observed with exploration in breadth, we conducted regression analyses that paralleled the exploration in breadth age ranges. Similar to exploration in breadth, age differences for exploration in depth were also observed in both the 18–47 and 43–72 age range analyses, *B* = −0.18, SE = 0.07, *p* < 0.01, and *B* = −0.33, SE = 0.05, *p* < 0.001, until the 68–72‐year‐old group (Panel B). However, no age effects emerged in the oldest cohort analyses, *B* = −0.18, SE = 0.07, *p* = 0.62. We also found a significant main effect for gender in the age 18–47 analyses, but the effect size was small, *B* = 0.06, SE = 0.03, *p* = 0.035. No other gender effects or age × gender interactions were observed in any analysis.

#### 6.2.3 Ruminative Exploration

We found significant main effects for age, with older adults reporting less ruminative exploration than younger adults. Additionally, women were more likely to report higher ruminative exploration overall, but this effect was small. The interaction effect was not significant (Panel C).

#### 6.2.4 Commitment Composite

Accounting for DIF, our analyses found no main effects for age or gender differences in commitment. However, we did find a significant age × gender interaction. For men, average commitment scores were reported across the sample. In contrast, women who were older reported lower commitment than younger women.

### 6.3 Associations Between Identity Processes and Satisfaction With Life Across the Lifespan

Our final set of analyses tested the degree to which identity processes were differentially associated with life satisfaction across the adult lifespan^8^. We first conducted correlational analyses in the overall sample, and then conducted a linear regression analysis, which regressed SWL scores on the four identity processes, age, gender, along with all 2‐way interaction terms between identity processes and both age and gender, and 3‐way interaction effects for age × gender × identity process^9^.

We first examined the correlations between identity processes and SWL scores (see Table S3 for correlations by age group). Commitment was positively associated with life satisfaction, *r* = 0.41, < 0.001, and the association appeared to be strongest in late‐middle adulthood. Both exploration in depth (*r* = 0.16, *p* < 0.01) and exploration in breadth (*r* = 0.05, *p* < 0.01) were also positively associated with SWL scores; however, the latter effect was trivial. In contrast, ruminative exploration was negatively associated with SWL scores in the overall sample, *r* = −0.26, *p <* 0.001.

**FIGURE 2:**
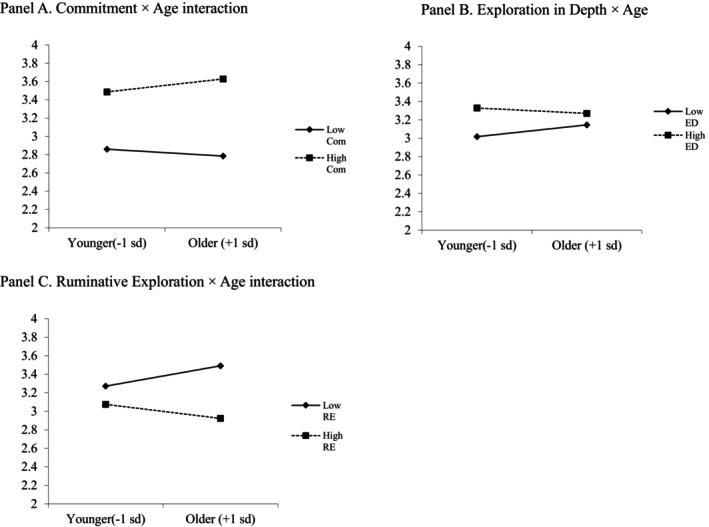
Moderation effects of age × identity process on SWL scores. (A) Commitment × age interaction; (B) Exploration in depth × age, and (C) Ruminative exploration × age interaction. Com, commitment composite; ED, exploration in depth; RE, ruminative exploration. Identity process variables mean‐centered, and ±1 SD represented.

Finally, we tested the degree to which age and gender moderate the associations between identity processes and life satisfaction (see Table 5). The model accounted for 24.6% of the variance in SWL scores, *F* (18,3829) = 69.11, *p <* 0.001. We found that ruminative exploration (−), exploration in depth (+), and commitment (+) uniquely contributed to explaining the variance in SWL scores. Notably, we found that age moderated these effects (see Figure 2). Specifically, for older adults, high commitment is related to greater life satisfaction, compared to younger cohorts with high life satisfaction (Panel A). We found a different pattern of results for the exploration variables. For exploration in depth (Panel B), we found that high exploration in depth was associated with greater SWL scores in younger participants, but this association was weaker for older participants. Finally, we found a somewhat reverse pattern for ruminative exploration (Panel C), in which older adults with low ruminative exploration report the greatest life satisfaction, and those older adults with high ruminative exploration report the lowest. We did not find any significant interaction effects between age and identity dimension. A main effect for gender was found, with women reporting greater satisfaction with life; however, this effect was small. No significant gender × identity process interactions were found, nor were any gender × identity process interactions.

**TABLE 5:** Regression analysis predicting satisfaction with life.

	B	SE	Std. Beta	Sig.
Age	0.001	0.001	0.014	0.383
Gender (1 = women)	0.066	0.030	0.036	0.027
Commitment	0.456	0.030	0.397	< 0.001
Exploration breadth	−0.046	0.034	−0.038	0.177
Exploration depth	0.124	0.027	0.122	< 0.001
Ruminative exploration	−0.212	0.027	−0.210	< 0.001
Age × Commitment	0.004	0.002	0.052	0.048
Age × Exploration breadth	−0.002	0.002	−0.022	0.445
Age × Exploration depth	−0.003	0.002	−0.053	0.039
Age × Ruminative exploration	−0.006	0.002	−0.092	< 0.001
Gender × Commitment	−0.081	0.045	−0.049	0.074
Gender × Exploration breadth	−0.071	0.050	−0.041	0.153
Gender × Exploration depth	0.029	0.039	0.020	0.460
Gender × Ruminative exploration	−0.038	0.038	−0.026	0.322
Age × Gender × Commitment	−0.001	0.003	−0.006	0.839
Age × Gender × Exploration Breadth	−0.001	0.003	−0.012	0.676
Age × Gender × Exploration depth	0.002	0.002	0.020	0.454
Age × Gender × Ruminative exploration	0.003	0.002	0.042	0.133

*Note: N* = 3830. Identity and age variables mean‐centered.

## 7 Discussion

Although the dual‐cycle identity approach suggests a dynamic pattern of identity‐related exploration and commitment across the lifespan, it is still unclear to which degree (a) age‐related differences can be observed, (b) there are gender differences in identity processes in different age groups, and (c) these dimensions may be associated with psychosocial well‐being during different life periods in the same or different ways. We examined these questions with a large community sample, first assessing the degree to which our identity measurement (the DIDS; Luyckx et al. 2008) was invariant across the adult lifespan. Whilst our analyses did not demonstrate full measurement invariance over the entire adult lifespan, they did indicate that invariance was achieved across large adjacent age ranges, which allowed us to make age‐related comparisons. Central to our study, age differences manifest for all types of exploration (i.e., in breadth, in depth, and ruminative), with lower exploration being reported in older age groups. However, our analyses suggest a more complex pattern for commitment. Although no age differences in commitment were observed between early and middle adulthood, we did observe that older individuals reported lower commitment between middle to late adulthood. Additionally, we found that commitment and exploration in depth were consistently positively associated with life satisfaction, whereas ruminative exploration negatively predicted life satisfaction scores. For exploration in breadth, the results were inconsistent across the age groups. Finally, although gender differences in identity processes were observed in some age groups, most notably for ruminative exploration, gender largely did not moderate the associations between age and identity processes; it also did not significantly moderate the association between identity and life satisfaction.

### 7.1 Differences in Measurement Invariance

To accurately assess age‐related differences in a sample with such a broad age range, a prerequisite and oft‐overlooked step is to establish measurement invariance. Overall, we found that all tested identity processes showed some degree of measurement invariance across the adult lifespan. However, we found a notable difference between the exploration in the breadth scale and the other scales. Specifically, we found evidence that both the commitment composite and ruminative exploration showed partial scalar invariance across the full sample. In contrast, exploration in breadth only demonstrated partial scalar invariance from 18 until about 47 years, then from 43 until 72 years, and again from age 68 until the oldest age group in our study. It should be noted that for the former two invariance tests, the same item “I think about different things I might do in the future”, needed to vary across age. Speculatively, this difference could arise from the interpretation of the word “things” as they relate to the “future”. Older individuals seem to more often endorse this item, which might represent a difference in temporal construal and an appreciation for their time remaining (Carstensen et al. 2024) in which younger participants think more abstractly about this item (e.g., “What are things I want to do in my life?”), whilst older adults construe this item more concretely (e.g., “What are things I want to do during the next few months?”). More broadly, we opine that the ruminative exploration scale showed the greatest degree of measurement invariance due to its overlap with negative affective traits such as neuroticism, which have also been found to be age‐measurement invariant (e.g., Mõttus and Rozgonjuk 2021).

### 7.2 Age and Gender Differences in Identity Processes

Contrary to our expectations, we did not find greater mean scores for the combined commitment dimension from younger to older individuals. At the surface, this finding contrasts with prior research on age‐related differences (Luyckx et al. 2013; Pulkkinen and Kokko 2000). However, it should be noted that those studies included different age ranges than the present one. For instance, Pulkkinen and Kokko (2000) only covered ages 27–36 years, albeit longitudinally. Similarly, Luyckx et al. (2013) included participants from 17 to 30, but only 10% of the sample was above 22 years of age, and only 2.8% were 28 years or older. Furthermore, we found modest evidence that these age‐related differences were moderated by gender, in which commitment was lower for women, but not for men. This finding may support an assertion made by Mehta et al. (2020), who suggest that a “career and care crunch” manifests during the period in which work‐life balance demands become the most prominent. This phenomenon may be experienced more greatly by women due to gender differences in societal expectations regarding care responsibilities (e.g., Löffler and Greitemeyer 2023) and could manifest as lowering career‐oriented commitments. This question spans beyond the current research, but future endeavors may benefit from parsing identity domains, such as career and home commitments (see Archer 1989), across this developmental period.

Results regarding the exploration dimensions were more aligned with our expectations. Firstly, ruminative exploration showed linear, negative age differences across the entire sample, consistent with prior research in negative emotionality, indicating that individuals on average show decreases in negative affectivity, neuroticism, and worry as they get older (Bleidorn et al. 2022; Gonçalves and Byrne 2013; Luyckx et al. 2013). Independent of age, women reported greater ruminative exploration across the entire adult lifespan, consistent with both adolescent and adult identity research (Luyckx et al. 2008, 2013, 2015). This finding is also supported by clinical research suggesting that women report ruminating more than men and may be especially susceptible to anxiety‐related cognitive factors (Johnson and Whisman 2013).

Additionally, our results largely align with Topolewska‐Siedzik and Cieciuch's (2019) study, which suggests that positive aspects of exploration (i.e., breadth and depth) were less likely to be reported over the lifespan. More broadly, our results resonate with Carstensen's (1993) Socioemotional Selectivity Theory (SST), which suggests that exploration goals become deprioritized in favor of more emotionally relevant goals. Moreover, our results correspond to Mehta et al. (2020)'s conception of *established adulthood* (i.e., the ages between 30 and 45), a period in which commitments become strongest as individuals experience increasing responsibilities in their careers. They may also make decisions about having children and perhaps providing care for aging parents. Our finding that gender moderates the relationship between age and exploration in breadth, with women reporting lower exploration toward the end of this age period, especially resonates with this point. As we did not find the predicted changes in commitment, but did find age differences in both positive and negative aspects of exploration, our work suggests that it is not so much commitment that increases, but that instead the reflection on, and questioning of, those commitments may decrease in this period of increased responsibilities. Note that increased responsibilities could either directly reduce the perceived viable option for exploration, or more indirectly affect exploration as the cognitive resources that were used for this earlier in life may now be taken up by these responsibilities. The idea that general resources available to an individual affect identity formation is a key premise of the identity capital model used to explain identity formation in younger age groups (Côté 2002).

### 7.3 Life Satisfaction

Across individuals of all ages, life satisfaction was positively associated with commitment and exploration in depth, and negatively associated with ruminative exploration. Given the dearth of supporting literature, we initially proposed a norm‐based hypothesis in which the strength of associations between identity processes and life satisfaction would be strongest in periods that coincided with age‐related identity norms for exploration (e.g., 20s–30s and retirement) and for having strong commitments (30s and older). However, our results were mixed. Supporting norm‐based assertions, our analyses suggested that exploration in depth was more strongly associated with life satisfaction for younger participants, and less so for older cohorts. Additionally, we found that ruminative exploration was descriptively more strongly associated with greater life satisfaction for older adults. Thus, life satisfaction seemed to be more strongly tied to those who reported greater ruminative exploration during a period in which having strong commitments and few intentions to change those may be perceived as the norm. Also, the commitment‐life satisfaction association seemed to be stronger in older adulthood, though this association was also apparent in younger adults.

These results are largely consistent with Luyckx et al. (2013), which found that correlations between depressive symptoms and identity processes, especially commitment making and exploration in breadth, were stronger during the late 20s, compared to adolescence. As individuals age, though, they may be concerned less about identity‐related exploration, which may be classified as “preparatory goals” (e.g., acquiring knowledge, exploring new directions in life; Carstensen 1993). This lessened concern may be reflected in the association of exploration in breadth and depth with life satisfaction being non‐significant in later adulthood. Instead, older adults tend to focus more on a sense of belonging, feelings, and emotional satisfaction. Similarly, Erikson (1950/1993) suggested that older adults would be more concerned with ego‐integrity than younger individuals, with the flip side of that being despair. Thus, the norm may be to worry less and hold on more to one's commitments. Our findings seem supportive of these arguments by suggesting that low commitment and high ruminative exploration are particularly associated with lower life satisfaction in older age groups. It should be noted that life events may impact both identity commitments and life satisfaction independently. Such cases might represent situations in which strong commitments are diminished, even if the individual desires to maintain them. These effects may exist in older age (e.g., related to bereavement or physical illness), but also in other ages (e.g., related to divorce or job loss).

Lastly, gender was examined as a moderator of the association between age and life satisfaction. Our results suggested no meaningful effects of gender, either as a main effect or a moderator of identity processes. Although we observed a significant association between gender and ruminative exploration, ruminative exploration appeared to be associated with life satisfaction similarly for men and women across the adult lifespan.

### 7.4 Directions for Future Research

Although our results provide promising evidence that explains the degree to which identity processes change over time, we must acknowledge several limitations. Most obvious is the use of a cross‐sectional study to test for age differences. Although cohort differences can illuminate potential effects of aging, they cannot substitute for longitudinal designs. For instance, parenting practices, social norms regarding exploration and commitment, gender differences in all of these, and many other cohort differences may exist, which may be reflected in our results (Baltes 1987). With respect to our results linking identity with life satisfaction, a longitudinal design would have the potential to examine the theoretically proposed dynamic interplay between commitment and exploration, and predictors of such fluctuations. It could also help to illuminate the degree to which age‐related changes in life satisfaction may co‐occur with changes in identity processes. Further, the design thus limits our ability to make conclusions regarding how these dimensions may change over time in general, not to mention the degree to which heterogeneity in such trajectories may exist. Such heterogeneity is important to identify, as research on different constructs has shown that specific trajectories can be risk factors for maladaptive behaviors, such as substance use and antisociality (e.g., Garofalo et al. 2024; Weller et al. 2021).

We must acknowledge that the sample was relatively homogeneous with respect to race and ethnicity. Whilst we made efforts to sample based on U.K. Census estimates for age, gender, and region, 81.7% of residents report white racial identity. Our sample was roughly in line with this figure, albeit slightly over it. Thus, issues regarding generalizability need to be evaluated in the context of future investigations, with more diverse samples. This being said, our sample included a large range of educational attainment and household income level, indicative of differences in social classes which also may relate to identity development, with even potentially stronger effects in majority populations, as is the case in the current study (e.g., Aries and Seider 2007; McAvay and Safi 2023).

In addition, we used the same measure across age groups, which has the advantage that it allows for direct comparisons of mean scores if measurement invariance is established (which was at least partially the case in this study). However, identity processes might present themselves in slightly different ways across age groups, with dimensions capturing readjustment and maintenance of identity commitments potentially being relevant in very old age (Kroger 2002). As the DIDS measure was designed and validated in the context of research on adolescents and younger adults (Luyckx et al. 2008), the items likely do not fully reflect exploration‐ and commitment‐related thoughts, feelings, and behaviors that adults may experience as they age. Thus, the DIDS‐SF may not fully capture the nuanced decision‐making that an individual experiences over the adult lifespan, nor may it capture shifts in meaning over time as an individual is presented with different opportunities and challenges related to careers and families, for instance. As a caveat, developing scales with such sensitivity to age‐specific elements would have consequences as well, because the use of different items for different age groups would mean that constructs are no longer directly comparable.

Moreover, in later adulthood, unique challenges (e.g., retirement, changes in family status) may impact an individual's sense of self, and factors such as changes in physical functioning can play a role in this (Lodi‐Smith and Roberts 2010). Naturally, capturing all of these decisions and events across the entire adult life span and the covariates that may predict them would extend beyond the scope of a single study. However, we hope that the current study sparks future research aimed at addressing these issues, albeit across narrower age ranges. In addition to longitudinal work, future studies could employ a qualitative design (e.g., interviews analyzed with, for example, thematic analysis) to examine whether there are such age‐specific manifestations of identity and what they look like.

Related to the previous points, the version of the DIDS we used only focuses on the domain of future orientation. Previous longitudinal research on identity formation in adulthood (Fadjukoff et al. 2016) found different developmental trajectories for different identity domains. Specifically, for men, changes in the religious domain went in the opposite direction compared to other domains such as politics and occupation. Versions of the DIDS tapping into identity domains other than future orientation are available (e.g., career plans and romantic relationships; Luyckx et al., 2014), and others could be developed. Hence, differential age trends across identity domains could be explored in future research using an alternative version of the DIDS.

Further, the dual‐cycle approach posits non‐linearity in the expression of the identity processes over time, which could provide future opportunities to examine covariates that may not only precede, but also coincide with and follow elevations in exploration and commitment. Related, the dual‐cycle model approach emphasizes the dynamics between identity processes cross‐sectionally in addition to longitudinally. A group‐differential approach (e.g., constructing identity clusters based on the DIDS dimensions, e.g., Verschueren et al. 2017) would be needed to capture the co‐occurrence of different dimensions and would give more insight into the adaptiveness of identity (e.g., whether individuals are exploring in the presence or absence of existing commitments). However, our current approach has some advantages over an identity‐cluster‐based approach, especially for age‐based comparisons. Specifically, age differences in the prevalence of identity clusters can be hard to interpret as subtle mean‐score differences in the underlying dimensions may be overlooked. Also, the theoretical meaning of clusters with the same name across different studies varies (e.g., Negru‐Subtirica and Klimstra 2021), whereas mean‐score differences between age groups can be interpreted less ambiguously.

### 7.5 Conclusion

Overall, the present study provides a comprehensive overview of age differences in identity processes across the adult lifespan. Although previous research has examined adult identity development, large‐scale examinations of age trends using the same measure across the adult lifespan had not previously been conducted. Our findings suggest that such an approach is feasible from a measurement perspective and that it can yield intuitively sound and conceptually important findings. We hope that the present study will inspire more research on identity processes beyond the typically studied age groups of adolescents and young adults.

## Author Contributions

All authors contributed to the scientific direction of the paper, including study development, writing and editing. The data collection and data analysis were conducted by Joshua A. Weller. Discussion and interpretation, as well as presentation of the data, was conducted by Elisabeth L. de Moor, Joshua A. Weller, and Theo A. Klimstra.

## Conflicts of Interest

The authors declare no conflicts of interest.

## Supporting information


Data S1.

## Endnotes

^1^
The instrument used in the Topolewska‐Siedzik and Cieciuch (2019) study focuses on “modes” which are only somewhat associated with the dimensions that we focus on, as they represent a blend of work focused on identity styles (Berzonsky et al. 2013), identity formation based on the Utrecht‐Management of Identity Commitments Scale (U‐MICS; Crocetti et al. 2008) and the instrument we will use: the DIDS (Luyckx et al. 2008; see Topolewska and Cieciuch 2017).

^2^
Preregistration can be found at osf.io: https://tinyurl.com/ywhpw8vj. This pre‐registration was made in conjunction with a larger project relating to gambling behavior and recruited a sample of individuals with past gambling involvement. We do not use this separate sample, nor do our analyses involve retrieving identity status classes through Latent Profile Analysis. Additionally, the age groups that we stated in the pre‐registration differ from what we report. However, as a robustness check, analyses using these age groups did not yield results that would meaningfully alter our findings or their interpretation. Further, based on reviewer suggestions, we tested measurement invariance of age, measured by a continuous variable, rather than age group analyses as stated in the pre‐registration. Thus, we consider this pre‐registration to be valid for the current study with respect to data‐cleaning and procedures pertaining solely to invariance testing of identity dimensions, and have included the pre‐registered results on OSF.io. However, the main analyses with respect to age‐differences in identity processes, and the associations between identity and well‐being were not explicitly covered by the preregistration. Datasets, syntax, and outputs are available on OSF: https://tinyurl.com/5n8v5rpt (Weller et al. 2025).

^3^
Because of the small number of those reported gender other than men/women, we restrict our analyses to men‐women comparisons when we test for gender differences.

^4^
Two items from the DIDS‐SF were omitted from the survey due to human error. The items that were not included were similar to retained item, though. Specifically, for exploration in depth, the omitted item was “I think about whether the aims I already have for life really suit me” compared to the retained: “I think about whether my future plans match what I really want.”, and for Identification with Commitment, the omitted item was “I sense that the direction I want to take in life will really suit me”, compared to the retained “I am sure that my plans for the future are right for me.”

^5^
Although the original SWL measure contained 5‐items, we omitted (“If I could live my life over, I would change almost nothing”, traditionally item 5) due to time constraints. Research supports that this item shows the weakest contribution to the total SWL score, and has correlated error variance with item 4 (“So far I have gotten the important things I want in life”; Clench‐Aas et al. 2011; Glaesmer et al. 2011; Hanzlová 2022; Hinz et al. 2025). Additionally, we tested the measurement invariance of the 4‐item measure across age groups. We found evidence for partial scalar invariance for the SWL, by relaxing one item. Results can be found at https://tinyurl.com/5n8v5rpt.

^6^
We chose not to create a composite for exploration in a similar manner based on theoretical and empirical reasons. For example, for the two exploration dimensions research suggests that there are more distinct age‐related patterns than there are for the two commitment dimensions (Luyckx et al. 2013).

^7^
The configural model was tested in this manner because it can only be tested with a multiple group design, as configural invariance is assumed in MNLFA.

^8^
MNLFA was conducted for the SWL scale. Our analyses found partial metric and scalar invariance for age. Results can be found on osf.io at https://tinyurl.com/5n8v5rpt.

^9^
We also tested for quadratic age effects, however, no significant effects were found. Thus we do not discuss this issue further.
