# Supplementary material for: Age Patterns in Dual‐Cycle Identity Processes and Their Associations With Life Satisfaction
Source: J Pers. 2025 Jun 26;94(2):304–19. doi: 10.1111/jopy.70001 (PMC12988339; doi:10.1111/jopy.70001)
Supplement: Supplementary file 1 — Data S1. [file JOPY-94-304-s001.docx]

Table SI-1. Age-group based DIF results for Age invariance (MNLFA), exploration in breadth.

| **Scale** | **DID-SF Item** | | | | |
| --- | --- | --- | --- | --- | --- |
| *Exploration in Breadth* | *Item 1* | *Item 4* | *Item 10* | |  |
| *Age 18-47* |  |  |  | |  |
| **Loading DIF (SE)** | .05(.02) | -.06(.03) | .02(.03) |  |  |
| **Intercept DIF (SE)** | -.03(.02) | .08(.02) | -.08(.02) | |  |
|  |  |  |  | |  |
| *Ages 43-72* |  |  |  | |  |
| **Loading DIF (SE)** | .01(.02) | -.01(.02) | .02(.02) | |  |
| **Intercept DIF (SE)** | -.01(.02) | .12^**^(.02) | -.10^*^(.02) | |  |
|  |  |  |  | |  |
| *68 and older* |  |  |  | |  |
| **Loading DIF (SE)** | .08(.10) | -.05(.04) | .08 (.07) | |  |
| **Intercept DIF (SE)** | -.03(.06) | -.03 (.04) | -.03(.07) | |  |
|  |  |  |  | |  |

Table SI-2.

*Configural model results for commitment composite, standardized factor loadings*

|  | **DIDS-SF Item** | | | | |
| --- | --- | --- | --- | --- | --- |
| Age Range | 2 | 3 | 5 | 8 | 11 |
| 18-22 | 0.79 | 0.78 | 0.64 | 0.47 | 0.57 |
| 23-27 | 0.8 | 0.79 | 0.72 | 0.64 | 0.58 |
| 28-32 | 0.75 | 0.85 | 0.77 | 0.74 | 0.63 |
| 33-37 | 0.77 | 0.78 | 0.74 | 0.64 | 0.49 |
| 38-42 | 0.76 | 0.83 | 0.76 | 0.76 | 0.73 |
| 43-47 | 0.77 | 0.87 | 0.79 | 0.77 | 0.75 |
| 48-52 | 0.73 | 0.82 | 0.75 | 0.81 | 0.77 |
| 53-57 | 0.82 | 0.87 | 0.81 | 0.78 | 0.76 |
| 58-62 | 0.72 | 0.89 | 0.79 | 0.73 | 0.75 |
| 63-67 | 0.75 | 0.84 | 0.8 | 0.71 | 0.73 |
| 68-72 | 0.7 | 0.86 | 0.72 | 0.58 | 0.64 |
| 73-77 | 0.7 | 0.82 | 0.65 | 0.73 | 0.69 |
| 78+ | 0.64 | 0.79 | 0.65 | 0.76 | 0.65 |

*Note.* CFI =.966, TLI =.932, RMSEA =.099, SRMR =.032

Table SI-3. Correlations between Identity Status Dimensions and Satisfaction with Life, by Age Group.

|  | Exploration  in Breadth | Exploration in Depth | Ruminative  Exploration | Commitment  Composite |
| --- | --- | --- | --- | --- |
| Overall Sample | .05^**^ | .16^**^ | **-.26**^**^ | **.41**^**^ |
| 18-22 years (n=299) | .09 | .20^**^ | -.10 | **.32^**^** |
| 23-27 years (n=301) | .11 | .24^**^ | -.08 | **.39^**^** |
| 28-32 years (n=336) | .13^*^ | .20^**^ | -.17^**^ | **.38^**^** |
| 33-37 years (n=319) | .11^*^ | .21^**^ | -.17^**^ | .**34^**^** |
| 38-42 years (n=313) | .06 | **.25^**^** | -.24^**^ | **.38^**^** |
| 43-47 years (n=299) | .14^*^ | **.26^**^** | -.14^*^ | **.45^**^** |
| 48-52 years (n=338) | .03 | .14^**^ | **-.37^**^** | **.45^**^** |
| 53-57 years (n=314) | .20^**^ | **.34^**^** | **-.28^**^** | **.54^**^** |
| 58-62 years (n=330) | .02 | .16^**^ | **-.43^**^** | **.53^**^** |
| 63-67 years (n=381) | -.02 | .19^**^ | **-.32^**^** | **.41^**^** |
| 68-72 years (n=328) | .08 | .05 | **-.31^**^** | **.42^**^** |
| 73-77 years (n=206) | .01 | -.05 | **-.28^**^** | **.36^**^** |
| 78 years and older (n=106) | -.19^*^ | .01 | **-.37^**^** | **.31^**^** |
